# Single-cell transcriptomic atlas of goat ovarian aging

**DOI:** 10.1186/s40104-023-00948-8

**Published:** 2023-12-06

**Authors:** Dejun Xu, Shuaifei Song, Fuguo Wang, Yawen Li, Ziyuan Li, Hui Yao, Yongju Zhao, Zhongquan Zhao

**Affiliations:** Chongqing Key Laboratory of Herbivore Science, College of Animal Science and Technology, Southwest University, Chongqing, 400715 China

**Keywords:** Goat, Granulosa cells, Ovarian aging, Single-cell transcriptomic

## Abstract

**Background:**

The ovaries are one of the first organs that undergo degenerative changes earlier in the aging process, and ovarian aging is shown by a decrease in the number and quality of oocytes. However, little is known about the molecular mechanisms of female age-related fertility decline in different types of ovarian cells during aging, especially in goats. Therefore, the aim of this study was to reveal the mechanisms driving ovarian aging in goats at single-cell resolution.

**Results:**

For the first time, we surveyed the single-cell transcriptomic landscape of over 27,000 ovarian cells from newborn, young and aging goats, and identified nine ovarian cell types with distinct gene-expression signatures. Functional enrichment analysis showed that ovarian cell types were involved in their own unique biological processes, such as Wnt beta-catenin signalling was enriched in germ cells, whereas ovarian steroidogenesis was enriched in granulosa cells (GCs). Further analysis showed that ovarian aging was linked to GCs-specific changes in the antioxidant system, oxidative phosphorylation, and apoptosis. Subsequently, we identified a series of dynamic genes, such as *AMH*, *CRABP2*, *THBS1* and *TIMP1*, which determined the fate of GCs. Additionally, *FOXO1*, *SOX4*, and *HIF1A* were identified as significant regulons that instructed the differentiation of GCs in a distinct manner during ovarian aging.

**Conclusions:**

This study revealed a comprehensive aging-associated transcriptomic atlas characterizing the cell type-specific mechanisms during ovarian aging at the single-cell level and offers new diagnostic biomarkers and potential therapeutic targets for age-related goat ovarian diseases.

**Supplementary Information:**

The online version contains supplementary material available at 10.1186/s40104-023-00948-8.

## Background

The ovary is the most critical and complex female reproductive organ that provides steroid sex hormones and mature oocytes to maintain endocrine homeostasis and female fertility by supporting ovarian cell types such as granulosa cells (GCs) and theca cells [1, 2]. Upon activation, primordial follicles are recruited, and begin to develop into antral follicles. However, only a very small number of follicles mature and ovulate, and the majority of follicles undergo atresia [3]. The ovary is one of the most active organs in animals, and exhibits early-onset aging-associated dysfunction, and ovarian aging results a decline in functional follicle reserve and oocyte quality, which directly affects the fertility and endocrine homeostasis of female animals [4].

The ovary consists of numerous heterogeneous cell types. Among them, oocytes are surrounded by GCs and/or theca cells that form the basic functional unit of the ovary called follicles [5]. GCs play vital roles in the growth and development of oocytes by exchanging materials and energy with oocytes through gap junctions [6]. In addition to the decrease in the follicular pool with age, other aging-associated physiological changes include endocrine imbalance, ovulation disorder, and poor oocyte quality. However, the underlying mechanism remains unknown, and it is difficult to anticipate the fate of cell types during ovarian aging.

Goats in Southwest China have high fertility, especially Dazu black goats with excellent multi-litter performance [7], which are good experimental models to study ovarian function. However, due to the complex structure of the ovary, it is difficult to accurately reveal cell-type-specific changes in gene expression, particularly in follicles at different developmental stages in one ovary. The single-cell RNA sequencing (scRNA-seq) technique is widely used in heterogeneous tissues, and provides transcriptional profiles at the single-cell level [8]. It is now possible to identify cell types, uncover heterogeneity, and construct developmental trajectories, and as such, scRNA-seq is well suited for exploring the underlying mechanisms of early ovarian development and ovarian aging. For example, Pei et al. [9] used scRNA-seq to map transcriptional profiles in the yak ovarian cortex. Niu and Spradling [10] found that pregranulosa cells were differentiated into two distinct pathways for supporting follicle formation in the mouse ovary. A primate ovarian aging model has been used to reveal cell-type-specific alterations in gene transcription [11]. Although the molecular characteristics of ovarian aging have been revealed in mouse and primate models [11, 12], the genetic mechanisms of ovarian aging in goats are unknown. To reveal aging-associated transcriptomic atlas characteristics, we performed scRNA-seq of ovarian cells from newborn, young and aging Dazu black goats using the 10× Genomics Chromium platform. The purpose of this study was to identify the ovarian cell types, reveal the fate of ovarian cells, and focus on the developmental trajectories and transcriptional regulatory networks of GCs during ovarian aging, which may offer a potential therapeutic target for anti-aging.

## Material and methods

### Goat ovary sample collection and dissociation

The ovarian samples used in this experiment were collected from healthy at D1 (newborn, 1 d), Y2 (young, 2 years old), and Y10 (aging, 10 years old) Dazu black goats. Briefly, the samples were collected from three different goat ovaries per age group. Goat ovaries were isolated immediately, rinsed three times with phosphate-buffered saline (PBS) to eliminate surface blood, and then cut into pieces with a scalpel. Then, the fresh ovary blocks were stored in tissue preservation solution (the reagent inhibits RNase and maintains the stability of tissue RNA, Beyotime, Shanghai, China), and immediately transferred to the laboratory for single-cell dissociation within 4 h. The ovarian fragments were further cut in DMEM/F12 medium containing 0.04% bovine serum albumin (BSA) with a sterile enzyme free scalpel. Ovarian fragments were then digested overnight at 4 °C in 0.25% trypsin–EDTA containing 1 mg/mL type II collagenase, followed by termination of digestion with 10% fetal bovine serum (FBS). Next, the dissociated cells were centrifuged for 5 min (1,000 r/min) and resuspended in a prewarmed DMEM/F12 culture containing 1% penicillin–streptomycin and 10% FBS at 37 °C. Cell debris was removed by centrifugation at a speed of 200 × *g* (5 min) for subsequent experiments. For each age stage, ovarian samples were prepared separately until finally pooled together for single-cell barcoding. The combined samples for each age group were sequenced once for scRNA-seq.

### Single-cell RNA sequencing

Before sequencing, the dead cells were removed from the cell suspension to meet the requirement that the number of living cells reached more than 85%. The qualified cells were washed and resuspended to prepare the appropriate cell concentration of 700–1,200 cells/µL for the 10× Genomics Chromium™ system. Then, single-cell mRNA libraries were generated using the Single-Cell 3’ Reagent V3 Kit (10× Genomics, Pleasanton, CA, USA) according to the manufacturer’s protocol. After gel bead in emulsion (GEM) generation, the reverse transcription reactions were barcoded using a unique molecular identifier for labelling, and then the cDNA libraries were amplified by PCR with appropriate cycles. Subsequently, the amplified cDNA libraries were fragmented and sequenced on an Illumina NovaSeq 6000 (Illumina, San Diego, USA). The sequencing depth should reach more than 50,000 read pairs/cells.

### scRNA-seq data processing and analysis

After sequencing was completed, we used *CellRanger* software (version 3.0.2) to perform preliminary processing on the original files. In brief, the raw BCL files generated by Illumina NovaSeq 6000 sequencing were demultiplexed into fastq files through the *CellRanger* mkfastq function, and the fastq file was processed to map the readings to the goat reference genome. The read-valid cell barcodes of low-quality cells were filtered, and a counting matrix was generated by unique molecular identifiers (UMIs). Additional normalization was performed on the filtered matrix in Seurat to obtain normalized counts. Highly variable genes in individual cells were identified, and principal component analysis (PCA) was performed to reduce the dimensionality of the top 30 principal components. Cells were then clustered at 0.6 resolution and visualized in two dimensions using Uniform Manifold Approximation and Projection (UMAP) and t-distributed Stochastic Neighborhood Embedding (t-SNE).

### Cell type annotation

We automatically annotated the cell clusters using the *SingleR* package. Then we artificially annotated these clusters into different cell types based on the cell marker dataset, tissue location, biological functions, and *SingleR* annotation results. Specifically, we calculated the differential expression of each cluster using the ‘bimod’ test as implemented in the Seurat FindMarkers function. Genes with a log_2_ average expression difference > 0.58 and *P* value < 0.05 were identified as marker genes (specific high-expression genes that can mark cell types). The Seurat-Bimod statistical test was used to find differentially expressed genes between each group of cells and other groups of cells (FDR ≤ 0.05 and |log_2_ Fold Change| ≥ 1.5). Gene ontology enrichment analysis for these significant differentially expressed genes was performed by the TopGO R package, and KEGG pathway enrichment analysis was performed using the hypergeometric test in R. Significantly enriched GO terms and KEGG pathways were selected by a threshold FDR (adjusted *P*-value) ≤ 0.05. By annotating the cell types artificially, different cell types were identified according to their known marker genes.

### Maker gene selection for each cell type

The differential expression of genes implies diversity in cellular biological functions. Therefore, we used Seurat’s “Find All Markers” function to identify differentially expressed genes (DEGs) for differential expression analysis between cells in one cluster and cells in other clusters in the dataset. The minimum percentage of each feature expression in each cluster was set to 0.25. The highly expressed DEGs were considered marker genes distinguishing each cluster.

### Gene Ontology (GO) analysis

GO analysis determines the significant relationship between different genes and biological functions. In this experiment, we used the *ClusterProfiler* R software package to perform GO enrichment analysis of the highly variable genes detected in each cell cluster. Additionally, the symbol gene IDs were translated into Entrez IDs using the bitr function. The enrichment of differentially expressed genes was analysed according to the results of the significance of the difference test, indicating a *P* value ≤ 0.05 for significant gene enrichment.

### Enrichment analysis

Gene set enrichment analysis (GSEA) software was used to analyse gene sets according to the highest enrichment DEGs per cell type from newborn, young, and aging goat ovaries. The test gene set of the GSEA algorithm was accumulated at the top or bottom of these ordered gene vectors. Upregulated differentially expressed genes were at the top of the gene list, while downregulated differentially expressed genes were at the bottom. Gene sets were obtained from the *MSigDB* database. GSVA software was used for gene set variation analysis (GSVA), and the R package “heatmap” function was used to visualize the results.

### CytoTRACE and RNA velocity analysis

To analyse the differentiation status of ovarian cells, the differentiation specificity of each cell was visualized via CytoTRACE, which was used to predict the development potential and relative differentiated state of each cell. Consequently, we loaded CytoTRACE into the R package and ran the CytoTRACE function on the custom RNA-Seq dataset. Finally, the reduction in data dimensionality was visualized using t-SNE. To further verify the trajectory inference analyses in the GCs, we performed RNA velocity analyses because RNA velocity can be used to infer developmental directionality by distinguishing unspliced and spliced mRNAs. The reads of the unspliced intron sequence were obtained from BAM files. After that, the RNA velocity was calculated and visualized by *scvelo* (0.2.2).

### Construction of developmental trajectory

The pseudotime algorithm reconstructs the molecular state transitions of a continuous process by quantifying the gradual differentiation of the single-cell transcriptome. Therefore, we plotted pseudotime trajectories of ovarian GCs using the *Monocle2* package (v2.12.0). A heatmap of the signature genes and highly variable genes over pseudotime was generated by the “plot pseudotime heatmap” function. Pseudotime genes were divided into four expression patterns and GO analysis of each pattern was shown by *Toppgene* with a *P* value < 0.05. Then, data dimension reduction was performed using the *DDRTree* algorithm.

### Cell cycle analysis

The cell cycle state of ovarian cells was determined by using the *CellCycleScoring* function in Seurat. Briefly, single cells with high expression of G2/M or S phase marker genes were scored as G2/M or S phase, respectively. The single cell with no expression of the two categories was scored as G1 phase. The list of marker genes used to score the cell cycle phases for each single cell is shown in Additional file 1. The cell cycle distribution of ovarian cells from newborn, young and aging goats was visualized by using t-SNE.

### Transcription factor (TF) analysis

To identify the key transcriptional regulators during ovarian aging, the potential transcriptional regulators were identified using single-cell regulatory network inference and clustering (SCENIC) analysis. Motif enrichment analysis was performed for each coexpression module. Based on the matrix, only the motif enriched target genes of transcription factors were retained, while other genes were removed. TFs and their direct target genes were defined as regulons. The regulon activity in each cell was analysed using *AUCell* software (1.8.0). Then, *AUCell* scores were calculated, and a higher score indicates that this TF strongly regulates target genes. Then the identified regulons were visualized and quantified in ovarian cells using *Loupe Browser* (6.0).

### Immunofluorescence

The goat ovarian samples from the three age groups were fixed overnight in 4% paraformaldehyde at 4 °C. Then, the samples were transferred to 70% ethanol and embedded in paraffin. After sectioning, the samples were deparaffinized, rehydrated, boiled in sodium citrate, and blocked in 5% BSA. For immunostaining, paraffin sections were incubated at 4 °C overnight with primary antibodies (AMH, 1:200, Proteintech, China), followed by incubation with fluorescent secondary antibodies (1:500, Proteintech, China) for 1 h at room temperature, and finally counterstained with 4′,6-diamidino-2-phenyl-indole (DAPI, Beyotime, China). The sections were captured with a fluorescence microscope (Zeiss, Axo observer 3, Germany).

## Results

### Identification of goat ovarian cell types using single-cell transcriptomics

**Fig. 1:**
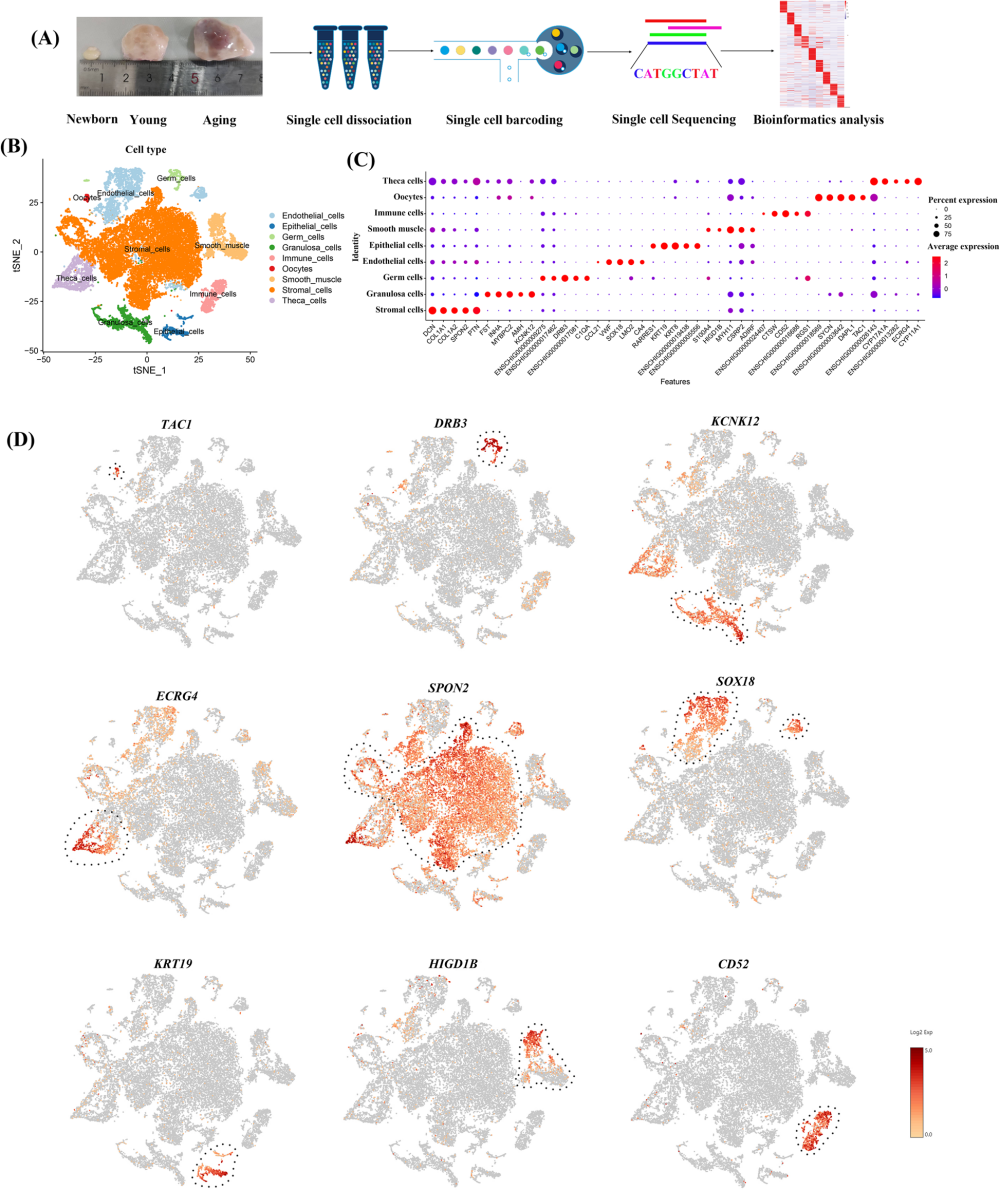
Identification of goat ovarian cell types by single-cell RNA-seq transcriptomics. **A** Flowcharts of goat ovarian scRNA-seq. **B** A t-SNE plot was used to visualize nine ovarian cell types. Each point corresponding to a single cell is colour-coded according to its cell type membership. **C** The dot plot shows distinct expression patterns of the selected signature genes for each cell type. **D** Expression specificity of the signature genes in ovarian cells, and the colour indicates the level of expression

To investigate the cell-type-specific transcriptional profiles during ovarian aging at single-cell resolution, ovarian samples were collected from newborn, young, and aging goats (Fig. 1A). The nFeature RNA, nCount RNA hemoglobin and percent mitochondrion successfully passed quality control (Additional file 2: Fig. S1A and Additional file 3). Following quality control, the high-quality transcriptomics of 27,049 single cells and a total of 1,427,619,277 reads were captured, with a median of 1,150 genes detected for each cell (Additional file 2: Fig. S1B and Additional file 4). To characterize ovarian cell types, the top 30 principal components (PCs) were chosen for cell clustering (Additional file 2: Fig. S1C). A t-SNE analysis was employed to cluster cells, and 23 transcriptionally distinct clusters were identified across three different ages (Additional file 2: Fig. S1D). The top 10 variable features of each cluster are shown in a heatmap (Additional file 2: Fig. S1E), indicating their expression specificity among the 23 clusters. The expression levels and percentages of the featured genes of interest across the 23 clusters are visualized in a dot matrix plot (Additional file 2: Fig. S1F).

Although the SingleR package provides a method to automatically annotate scRNA data, it seems unsuitable for use in goat cell types, because there are currently no reports on marker gene databases specifically for goat ovaries. In view of this, manual identification was performed based on the function of the marker genes in each cluster. To further identify these cell clusters, we mapped the gene-expression profiles of well-defined cell-type-specific markers in the t-SNE plot (Fig. 1B). Nine main cell populations were identified in the goat ovary, including oocytes, germ cells, GCs, theca cells, stromal cells, epithelial cells, endothelial cells, immune cells, and smooth muscle cells. By analysing the cluster-expressed genes, different cell types were identified according to their marker gene expression (Additional file 5). Briefly, Cluster 21 specifically expressed at high levels of the oocyte marker gene *FIGLA* [13]. Clusters 16 and 23 expressed high levels of the germ cell marker *PRDM1* [13]. Cluster 7, 18 and 20, were specifically expressed at high levels of the GC markers *AMH, FSHR* and *Fst* [13–15]. Furthermore, several important cell types were also identified including stromal cells (*PDGFRA* and *DCN*, Clusters 1, 2, 3, 4, 9, and 14) [13, 16], endothelial cells (*Cdh5* and *PECAM1*, Clusters 5, 8 and 17) [13], theca cells (*Cyp17a1A*, Clusters 6) [14], smooth muscle cells (*RGS5* and *TAGLN*, Clusters 10 and 11) [15], epithelial cells (*Krt19*, *CD24* and *DSP*, Clusters 15 and 19) [17], and immune cells (*Ptprc* and *CD69*, Clusters 12) [14]. Subsequently, we coloured the single cells according to the expression levels of several expected marker genes (Fig. 1D). The expression scores and percentages of cell type-specific genes were visualized in a dot matrix (Fig. 1C). It is worth noting that a series of cell type-specific expressed novel marker genes were identified in ovarian cells of goats (Fig. 1D), such as oocyte markers *TAC1* and *DAPL1*, germ cell markers *DRB3* and *C1QA*, GCs markers *KCNK12* and *MYBPC2*, theca cell markers *ECRG4* and ENSCHIG00000013282, stromal cell markers *SPON2* and *COL1A1*, endothelial cell markers *SOX18* and *LMO2*, epithelial cell markers *KRT19* and *KRT8*, smooth muscle cell markers *HIGD1B* and *MYH11*, and immune cell markers *CD52* and *CTSW*. Collectively, we identified nine different ovarian cell types, and discovered novel markers for goat cell types.

### Gene expression signatures of ovarian cells during aging

**Fig. 2:**
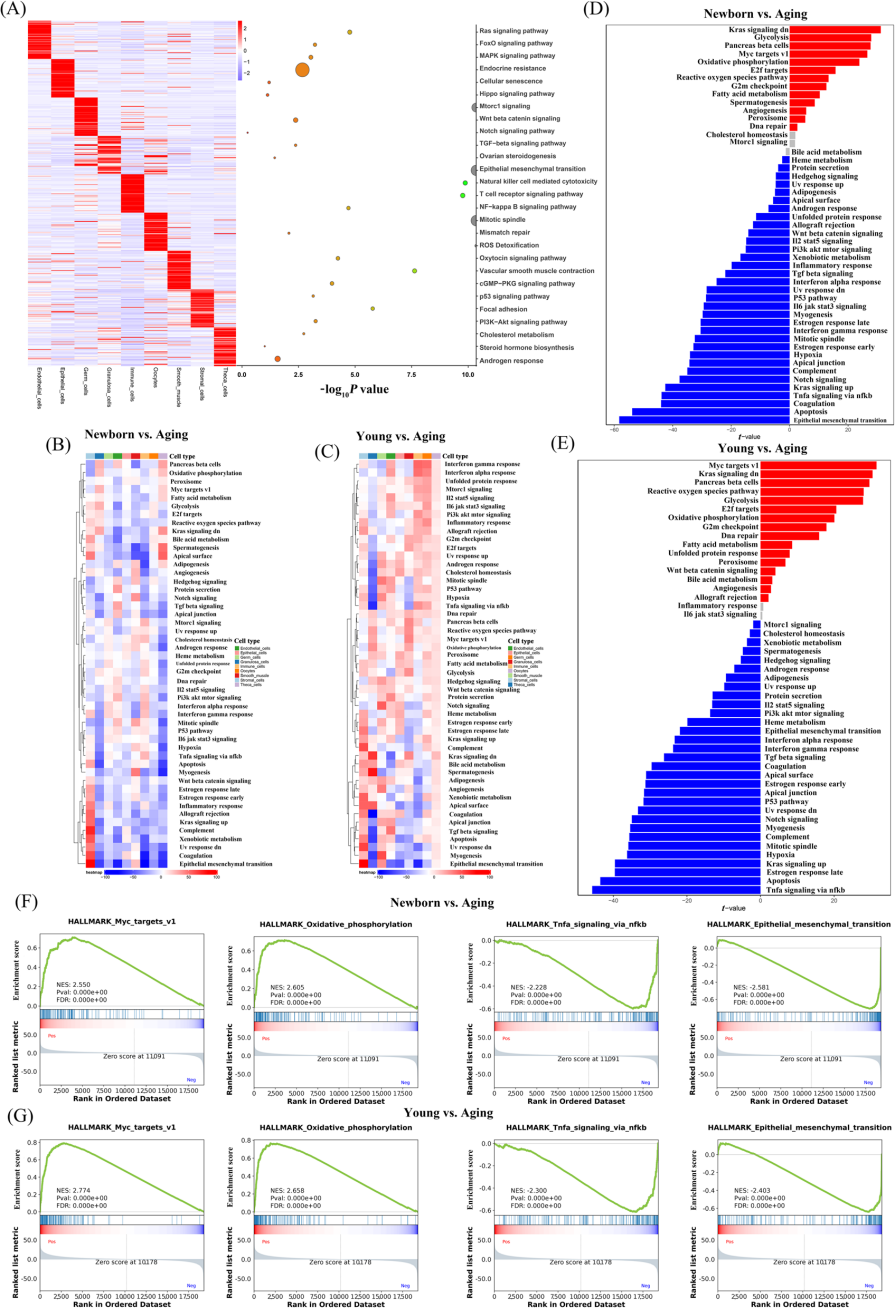
Gene expression signatures of granulosa cells during ovarian aging. **A** Left: heatmap showing the expression signatures of the top 50 specifically expressed genes in each cell type; the value for each gene is the row-scaled Z score. Right: representative GO terms for specific genes. **B** A heatmap was visualized based on the highest enrichment DEGs between newborn and aging goats in ovarian cells by GSVA. **C** A heatmap was visualized based on the highest enrichment DEGs between young and aging goats in ovarian cells by the GSVA. **D **and **E** The histogram showing the biological process terms from GSVA in ovarian granulosa cells. **F **and **G** The trends of biological terms were obtained by GSEA in newborn, young, and aging ovarian granulosa cells

After identifying major cell types, we then investigated the molecular changes at single-cell resolution during ovarian aging. As shown in Fig. 2A, three biological functions of the nine major cell types were produced among newborn, young, and aging goats by using GO analysis of the top 50 specific genes at each developmental stage, revealing unique characteristics of ovarian cells. For example, GO terms specific to oocytes included “mitotic spindle”, “mismatch repair” and “ROS detoxification”, suggesting that aging-associated ROS signalling may be associated with nuclear maturation of oocytes. GO terms including “mTOR1 signalling”, “Wnt beta-catenin signalling” and “Notch signalling pathway” were enriched for germ cells. GCs are involved in the “TGF-beta signalling pathway”, “ovarian steroidogenesis” and “epithelial mesenchymal transition”. GO terms including “cholesterol metabolism”, “steroid hormone biosynthesis” and “androgen response” for theca cells indicated that GCs and theca cells provide hormones for follicular development. GO terms including “Ras signalling pathway”, “FoxO signalling pathway” and “MAPK signalling pathway” for endothelial cells. The specifically expressed genes at highly expressed in epithelial cells mainly participate in “endocrine resistance”, “cellular senescence” and the “Hippo signalling pathway”. Immune cells tend to be involved in the “Natural killer cell mediated cytotoxicity”, “T cell receptor signalling pathway” and “NF-kappa B signalling pathway”. GO terms including “Oxytocin signaling pathway”, “Vascular smooth muscle contraction” and “cGMP-PKG signalling pathway” for smooth muscle. Stromal cells are mainly involved in “p53 signaling pathway”, “Focal adhesion” and “PI3K-Akt signalling pathway”. Collectively, GO enrichment shows that each cell type of the ovary is involved in a unique biological process.

To reveal the changes in gene expression signatures of cell types during ovarian aging, following GSVA, a heatmap was visualized based on the highest enrichment DEGs per cell type from newborn, young and aging goat ovaries. Some important biological processes, such as “epithelial mesenchymal transition” and “Uv response dn”, were upregulated in most types of aged ovaries, including endothelial cells, epithelial cells, GCs, immune cells, and theca cells of aging ovaries compared with those of young, newborn goats, whereas these gene expression signatures were downregulated in Sertoli cells (Fig. 2B and C). Compared with young goats, “androgen response” and “cholesterol homeostasis” were downregulated in other cell types of aging ovaries, but not in theca cells. It is worth noting that development-related “Wnt beta catenin signalling” and “Hedgehog signalling” are downregulated in most ovarian cells. Moreover, “oxidative phosphorylation”, “fatty acid metabolism” and “glycolysis” were decreased in aging ovaries, suggesting that aging causes a decline in the metabolic function of ovarian cells. To analyse gene-expression changes involved in aging during folliculogenesis, we identified biological processes of GCs in comparisons between different ages. Compared with newborn and young goats, the biological processes involved in “Myc targets”, “Kras signalling dn”, “Glycolysis”, “Reactive oxygen species pathway” and “Oxidative phosphorylation” were downregulated, whereas “Tnfa signalling via nfkb”, “Apoptosis”, “Epithelial mesenchymal transition”, and “Kras signalling up” were upregulated in aged GCs (Fig. 2D and E). GSEA also highlighted the most significant negative enrichment for genes of GCs upregulated in “Myc targets”, “Oxidative phosphorylation” and positive enrichment for genes downregulated in “epithelial mesenchymal transition” and “TNFa signalling via nfkb” in aged goats compared with those of newborn and young goats. The changes in the gene expression signatures of GCs revealed that “Myc targets” and “Oxidative phosphorylation” signallings were inhibited, whereas “epithelial mesenchymal transition” and “TNFa signalling” were activated in ovarian aging, suggesting that ovarian aging is closely related to the stagnation of GC proliferation and differentiation and the decline in mitochondrial function, cellular immunity and inflammation.

### scRNA-seq reveals the fate of GCs during ovarian aging

**Fig. 3:**
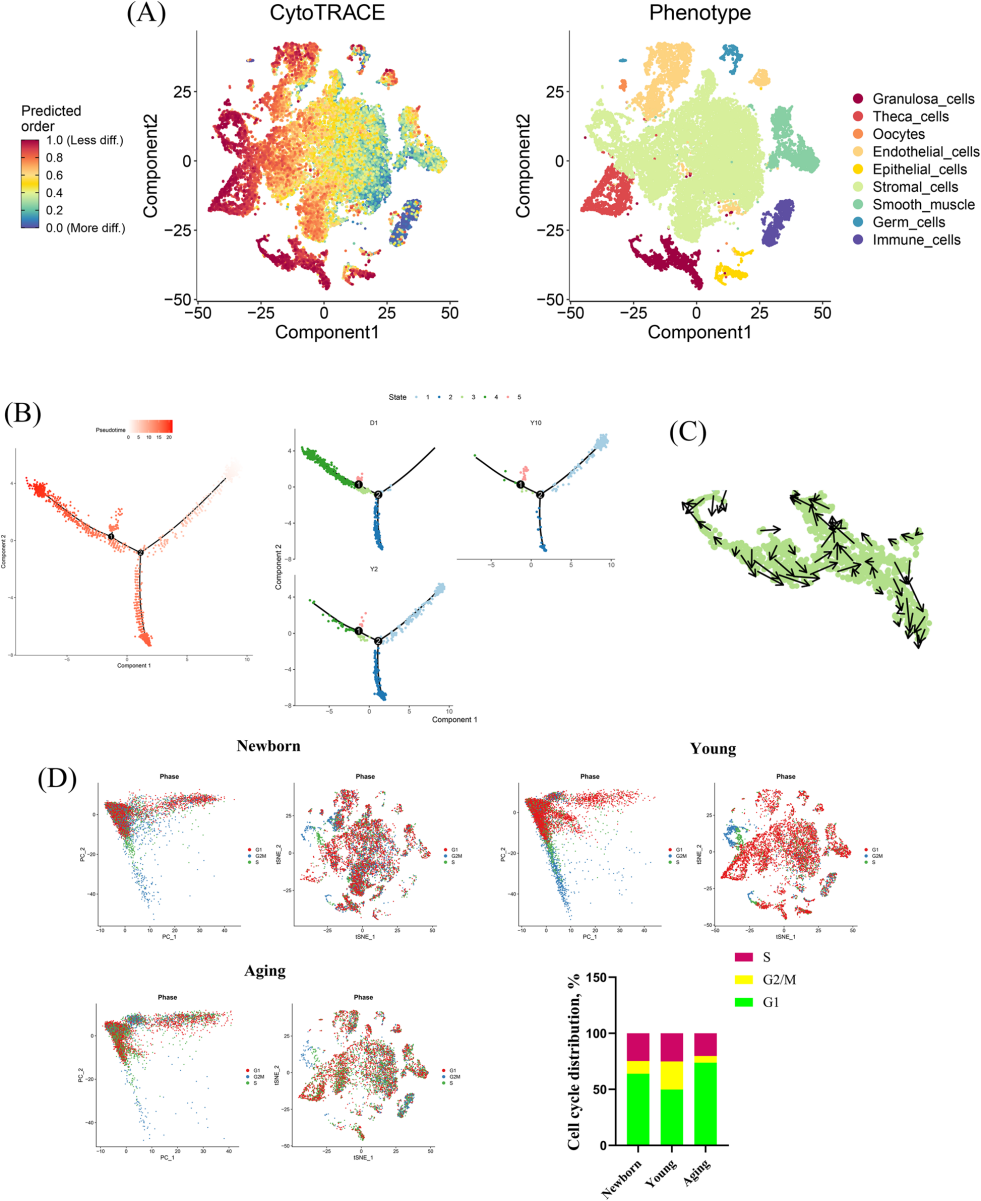
scRNA-seq reveals the fate of GCs during aging. **A** The differentiation potential of ovarian cells was visualized by CytoTRACE analysis. The differentiation capacity from less to more is indicated by a gradient colour from red to blue. **B** Scatterplot showing the differential trajectories of GCs in newborn, young and aging goats with a pseudotime scale by Monocle analysis. **C** The developmental trajectory of GCs was shown by RNA velocity analyses. Arrows indicate the development direction of GCs. **D** The phase of the cell cycle was visualized in newborn, young and aging goats by using t-SNE. The proportion of GCs that were shown in each phase of the cell cycle

In the CytoTRACE analysis, the differentiation potential of ovarian cells was visualized with t-SNE. As shown in the plot, some ovarian cell types had a low degree of differentiation, such as GCs, theca cells, endothelial cells and epithelial cells, whereas germ cells, immune cells, and smooth muscle showed high differentiation potential, indicating that they are more functionally specific (Fig. 3A). The fate of GCs plays a key role in determining ovarian function. To reveal the temporal dynamics of GCs during aging, the development trajectories were constructed by Monocle analysis. For GCs, the pseudotime trajectory displayed two branch points, and the results clearly demonstrated the nonuniform development of GCs from primordial follicles to antral follicles (Fig. 3B). It is worth noting that most of the GCs in aged goats were present in state 1 and rarely present in state 4 (Fig. 3B). These GCs showed a time-ordered decrease over pseudotime, indicating that the differentiation potential of GCs is gradually lost during aging. We performed RNA velocity analyses to further verify the developmental trajectory in GCs. The results showed that most of the RNA velocity vectors of GCs had obvious branch and endpoint directions (Fig. 3C), which verified our trajectory inference analysis. To further identify the fate of ovarian cells during aging, we next determined the cell cycle state of each cell using the cell cycle scoring function of the Seurat package for R. The mitotic cell cycle consisting of G1, S and G2/M phases was successfully identified by using t-SNE. As shown in the plot, a high proportion of G1 phases was clearly present in GCs of the aged ovary (Fig. 3D). This finding further confirmed that a large number of GCs undergo cell cycle G1-phase arrest, resulting in irreversible cell proliferation arrest.

### Reconstruction of temporal dynamics of GCs during ovarian aging

**Fig. 4:**
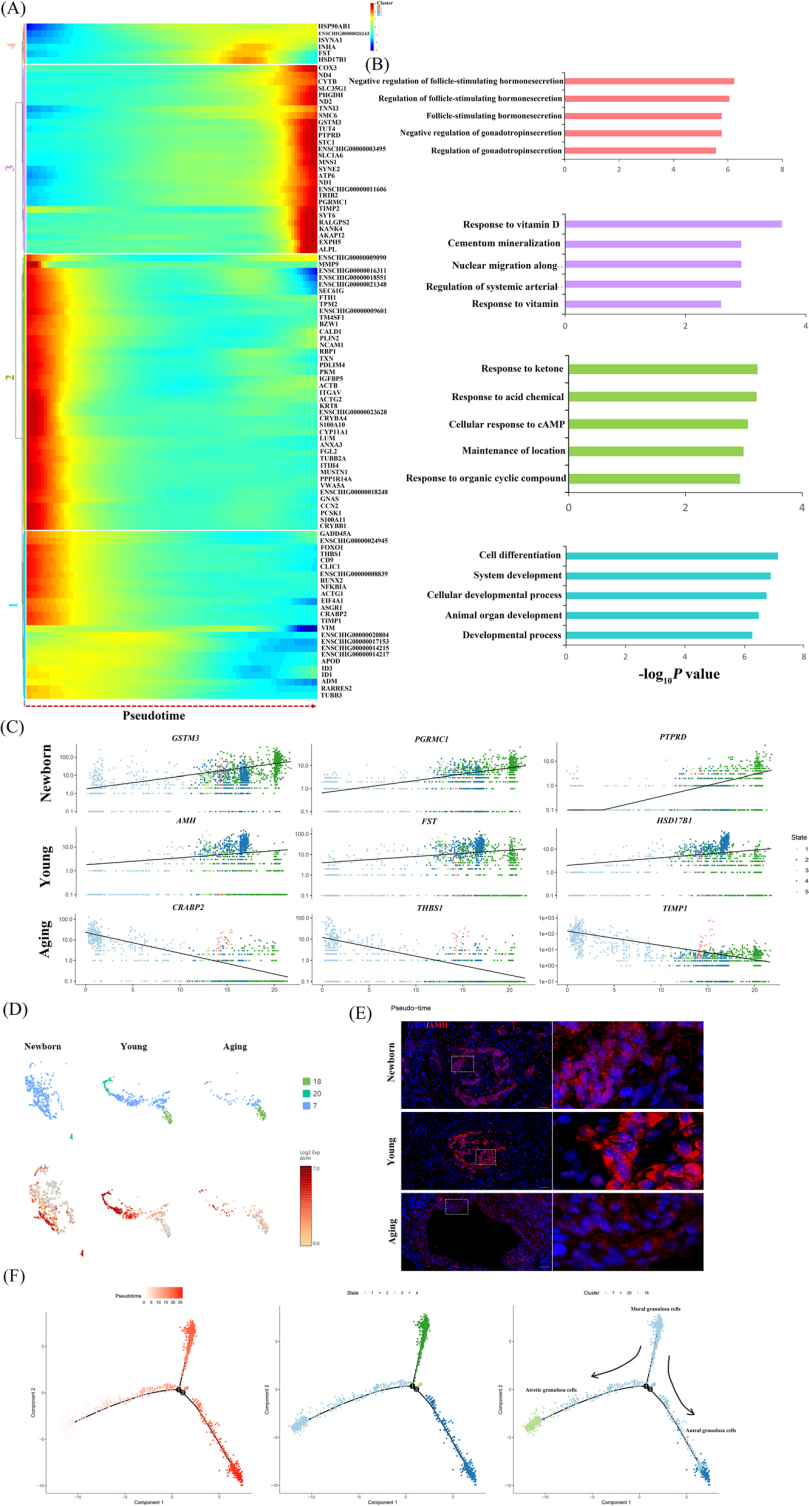
Pseudotime trajectory analysis delineated the temporal dynamics of GCs during ovarian aging. **A** Pseudotime heatmap showing dynamic gene expression profiles during GC fate commitment. The four gene sets were determined by *k*-means clustering according to their expression patterns. The expression level of dynamic genes from high to low is indicated by a colour gradient from red to blue. **B** The top 5 enriched GO terms for each gene set are shown based on the dynamic genes of GCs. The corresponding clusters' GO terms are represented in the same colour. **C** Visualization of expression trends of the signature genes over pseudotime in GCs. **D** Expression of AMH in GCs. The colour indicates the level of expression. **E** Immunostaining of follicles for *AMH.* Three different ovaries were immunostained in each age group. Scale bars are 20 μm. **F** Scatterplot showing the differential trajectories of three GCs subtypes over pseudotime by Monocle. Arrows indicate that the mural GC developed into atretic and antral GCs. State refers to the fact that during the development and differentiation of GCs, different genomes are expressed (some genes are activated, while others are silenced). Branch points refer to the changes in gene expression/the emergence of new cell types during the process of cell development and differentiation

After delineation of the trajectory inference, we focused on the temporal dynamics of GCs to reveal the changes in fate decisions during ovarian aging. The trends of pseudotime-dependent genes along the pseudotime timeline were classified into four clusters with different expression dynamics in a heatmap. As shown in the heatmap, the different genes in Cluster 1 and 2 appeared to be upregulated along the pseudotimeline axis, and genes in Cluster 3 and 4 showed the opposite trend (Fig. 4A). Gene functional enrichment analysis revealed that Cluster 1 genes were highly enriched in the GO terms “regulation of follicle stimulating hormone secretion” and “regulation of gonadotropin secretion” (Fig. 4B). Notably, *INHA*, *FST* and *HSD17B1*, which are involved in follicular development showed a tide-wave trend along the pseudotimeline, thus presenting a trend of expression levels increasing first and then decreasing (Fig. 4A). Cluster 2 genes showed a constantly upregulated trend along the pseudotimeline, such as *ALPL* and *STC1*, and GO terms were related with “response to vitamin”, suggesting that these gene dynamics play important roles in metabolic processes (Fig. 4A and B). In addition, we also identified several genes such as *CYP11A1*, *GNAS*, and *IGFBP5*, which are involved in multiple biological processes, including “response to ketone” and “cellular response to cAMP” in Cluster 3 (Fig. 4A and B). Specifically, *CYP11A1,* the rate-limiting enzyme of progesterone synthesis, is important for ovarian corpus luteum secretion [18], and its expression showed an obvious downwards trend along the pseudotimeline axis (Fig. 4A). Meanwhile, GO analysis enriched the generation of “cell differentiation”, “cellular developmental process” and “developmental process” in Cluster 4 (Fig. 4B). The activity of those biological processes was also inhibited along the pseudotimeline axis, indicating that follicular development gradually weakened.

Based on DEG analysis over pseudotime, we then analysed the gene dynamics of GCs at different ages. As expected, a series of dynamic genes of GCs showed increased expression over pseudotime in newborn goats, including *GSTM3*, *PGRMC1*, and *PTPRD* (Fig. 4C). These dynamic genes play important roles in the response to steroid hormones, or developmental processes. Meanwhile, some granulosa cell-specific genes such as *AMH*, *FST*, and *HSD17B1*, which reflect ovarian reserve were mainly enriched in young goats (Fig. 4C). Interestingly, dynamic genes, including *CRABP2*, *THBS1* and *TIMP1*, showed constantly downregulated trend along the pseudotime axis and are important for follicular development in aged goats (Fig. 4C). Since all the cell clusters had been successfully characterized, the relative expression of dynamic genes was then quantified in GC clusters (Clusters 7, 18, and 20). *AMH* is known to be highly expressed in growing follicles but not in atretic follicles. We also observed that *AMH* was abundant in growing follicles by immunostaining (Additional file 6). As shown in Fig. 4D, *AMH* was hardly expressed in sub-cluster 18, and Cluster 7 and 20 were abundant in newborn ovaries, while Cluster 18 was very few. Using immunostaining, we confirmed that the expression of *AMH* in GCs of aging goats was lower than that of young goats (Fig. 4E), indicating reduced ovarian reserve in aging goats. Based on pseudotime trajectory analysis, we further identified that cluster 7 was differentiated into Cluster 18, and 20. These evidences suggest that Cluster 7, 18 and 20 were mural GCs, atretic GCs and antral GCs, respectively. The data indicated that State 4 was at the early stages of follicle development, while States 1 and 2 were at the late stages of follicle development (Fig. 4F). It is worth noting that the expression levels of dynamic genes, including *AMH, FST* and *HSD17B1*, were downregulated in mural and antral GCs of aging ovaries, suggesting that the ovarian pool decreases with aging (Additional file 7). Interestingly, the *PTPRD* gene gradually was decreased with age in Clusters 7 and 20, whereas it increased in Cluster 18 to a high level of expression during aging (Additional file 7). Meanwhile, *PTPRD* was highly expressed in the early stage of GCs. It is well known that *PTPRD* is a signalling molecule that regulates a variety of cellular processes, including cell growth, differentiation, and mitosis. The results showed that not all GCs had a decrease in their ability to differentiate with aging, in contrast, some GCs in mature follicles, such as Cluster 18, had an enhanced differentiation ability, resulting in the formation of branch points during follicular development. Furthermore, dynamic genes associated with differentiation, including *CRABP2*, *THBS1* and *TIMP1*, were highly increased in GCs of aging goats compared with those of young goats (Additional file 7). Although the expression of these dynamic genes was low in young growing follicles, they increased rapidly and subsequently decreased along the pseudotimeline axis in aging growing follicles. In total, we recaptured the sequential and stepwise trajectory of GC development, and identified a series of pseudotime-dependent genes that may play a role in disorders of follicular recruitment and development in ovarian aging.

### Transcriptional regulatory networks of GCs during ovarian aging

**Fig. 5:**
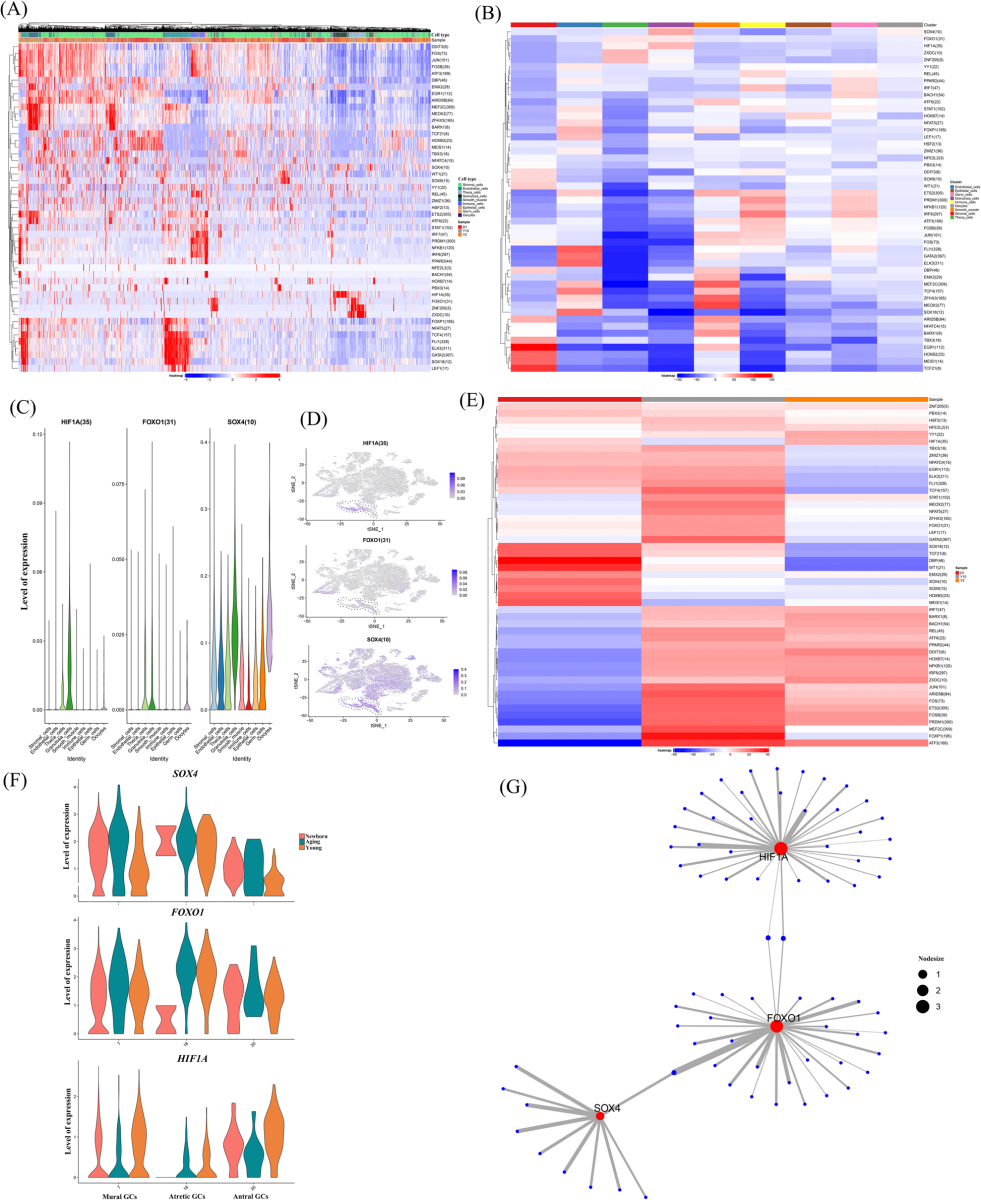
Transcriptional regulatory networks of GCs during ovarian aging. **A** A heatmap visualized the significant regulons by SCENIC analysis in ovarian cells. The score of regulation from high to low is indicated by a colour gradient from red to blue. The number in parentheses indicates the number of target genes regulated by this transcription factor. **B** A heatmap showing different TFs in ovarian cell types by *t-*test. Red indicates a larger *t-*value, and blue indicates a smaller *t-*value. **C** Violin plot showing feature TF expression in ovarian cells. **D** t-SNE plots showing the expression of TFs in GCs. The colour indicates the level of expression. **E** A heatmap demonstrating different TFs in newborn, young and aging goats by *t-*test. **F** Violin plot showing expression of *SOX4*, *FOXO1* and *HIF1A* in GC subtypes from newborn, young and aging goats. **G** Regulatory network visualizing potential key TFs in downstream target genes. The dot size represents the regulatory weight of TFs

To further explore the master regulators of follicle aging, we analysed the differentially expressed TFs in the ovaries of newborn, young, and aging goats. A heatmap of the AUC scores of TF motifs was visualized by SCENIC analysis in the main cell types. As shown in the plot, we identified 59 significant regulons that regulated ovarian gene expression patterns (Fig. 5A and Additional file 8). It is worth noting that *HIF1A*, *EMX2* and *SOX4* motifs were highly activated in GCs (Fig. 5B). *HIF1A* was found to be associated with oxygen sensors. *EMX2* is a driver of development in the female reproductive system. Similar to the previous GO enrichment in “epithelial mesenchymal transition”, *SOX4* has profound roles in the transcriptional regulation of this biological process. Subsequently, we compared the differential transcription factors for ovarian cell types. We found that transcription factor genes such as *FOXO1*, *SOX4*, and *HIF1A* were highly expressed in GCs (Fig. 5C and D). In particular, *FOXO1* and *SOX4* were specifically present in GCs and theca cells, which might play an important role in follicular development. Based on SCENIC analysis of ovaries in different aged groups, *HIF1A* was downregulated, whereas *FOXO1* exhibited peak expression in aged ovaries (Fig. 5E). Moreover, hierarchical cluster analysis of these transcription factors showed that *FOXO1* expression levels did not change with age in GCs (Fig. 5E). In addition to atretic GCs, *SOX4* showed higher levels in both antral GCs and mural GCs of the aging group than in the newborn and young groups (Fig. 5E). For atretic GCs, *HIF1A* was hardly observed in newborn goats, whereas it was highly expressed in antral GCs and mural GCs (Fig. 5E). Notably, there was little change in *HIF1A* levels in atretic GCs, while *HIF1A* levels in antral GCs and mural GCs of the aging group were downregulated compared to those of the young group (Fig. 5E). Overall, these results suggest that the follicle development stages show different gene expression patterns, and *HIF1A* and *SOX4* may play a key role in the aging process. Furthermore, the regulatory network in GCs revealed that potential key TFs, including *HIF1A*, *SOX4*, and *FOXO1,* regulated downstream targets (Fig. 5F). These target genes such as *WNT5A*, *IGFBP2*, *FST* and *SCT1* (Additional file 9), are involved in the fate of GCs, and may contribute to the compromised proliferation arrest of GC responses in ovarian aging.

## Discussion

In this study, we present the first single-cell survey of ovarian aging in goats that provides new insights into the mechanisms by which transcriptional profiles change during aging. Different cell types have been identified in the human, monkey, mouse, and cattle adult ovaries, including GCs, oocytes, stromal cells, and immune cells [8, 16, 19, 20]. In the present study, we captured high-quality data to identify ovarian cells at the single cell level by using the 10× Genomics Chromium™ system. Here, nine main cell types were successfully identified including oocytes, germ cells, GCs, theca cells, stromal cells, epithelial cells, endothelial cells, immune cells, and smooth muscle cells. Consistent with previous reports, some marker genes such as *FIGLA, PRDM1*, *AMH*, *PDGFRA*, and *Cyp17a1A* were specifically expressed at high levels in oocytes, germ cells, GCs, stromal cells, and theca cells [10, 13, 14]. However, some markers, such as *DAZL*, *DDX4*, and *OCT4,* in germ cell of mice or humans were not found in goat ovaries, suggesting that the marker genes in goat ovaries are not completely conserved with those in other animals [21, 22]. Subsequently, we identified a series of cell type-specific expressed novel marker genes, including *DAPL1*, *DRB*, *MYBPC*, *ECRG4*, *COL1A1*, *SOX18*, *KRT8*, *MYH11* and *CTSW*, which provided valuable information to distinguish ovarian cell types of livestock animals.

Most previous reports have described detailed morphological alterations during ovarian aging in mammals, such as diminished follicle reserve, whereas the molecular mechanisms underlying ovarian aging remain largely unknown at the single cell level. Here, we show that ovarian cell types are involved in their own unique biological processes by using scRNA-seq. For example, Wnt beta-catenin signalling was enriched in germ cells, and GCs were involved in ovarian steroidogenesis. It is well accepted that Wnt signalling is required for follicle development, and steroid hormones synthesized by granulosa cells play an important role in maintaining female fertility [23]. Moreover, by comparing cell-type-specific and age-associated gene-expression changes in ovarian cell types in newborn, young and old goats, we found that ovarian aging is linked to GCs-specific downregulation of the antioxidant system, oxidative phosphorylation in mitochondria, and apoptosis. Supporting these findings, previous studies revealed that oxidative damage and mitochondrial dysfunction were observed in aged GCs through scRNA-seq analysis [10]. This further supports the previous theory that GC apoptosis is associated with follicular atresia, as aging ovaries have more atresia follicles [24]. Furthermore, we observed increased activation of TNFa signalling via nfkb in aged GCs, which was closely related to the inflammatory response, and may also take place in goat ovarian GCs during aging. Consistently, Fan et al. [16] demonstrated that GCs are involved in the immune response during follicular remodelling in the human adult ovary by using scRNA-seq. These results suggest that the inflammatory or immune response of GCs may be linked to ovarian aging and follicle function, and its role deserves to be explored in the future.

GCs play a role in determining endocrine homeostasis and oocyte development in the ovary. We found that GCs were less differentiated by CytoTRACE analysis, suggesting that the morphological structure and function of GCs may be more complex. Based on single-cell pseudotime trajectory inference, the number of pregranulosa cells is gradually depleted with age, especially in aging ovaries, which are almost depleted. The available pool of primordial follicles is determined by the proper proliferation and recruited number of pregranulosa cells [11]. Meanwhile, we observed that the GCs in aged goats showed irreversible proliferation arrest, exhibiting an ovarian aging phenotype. By analysing the expression of different genes over pseudotime, we identified a series of dynamic genes involved in follicular function. These dynamic genes were enriched in multiple biological processes, including the regulation of follicle stimulating hormone secretion, cellular developmental processes. It is worth noting that the current study revealed three clusters of GCs that displayed distinct features along pseudotime. Zhao et al. [19] reported that subpopulations of GCs play distinct roles in foetal ovary development. Interestingly, we found that some dynamic genes, such as *PTPRD*, were expressed at reduced levels in the mural and antral GCs, but at high levels in atresia GCs. It is well known that *PTPRD* is a signalling molecule that regulates multiple processes, including apoptosis, differentiation, and mitosis cycles [25, 26]. Similarly, GC functional genes, such as *AMH*, *FST* and *HSD17B1*, also showed distinct expression characteristics in subcluster GCs in this study. This evidence suggests that the fate and functions of subcluster GCs are inconsistent, and the distinct roles are largely determined by their gene expression patterns during ovarian aging. Specifically, *AMH*, *FST* and *HSD17B1* showed similar trends over pseudotime, and were highly expressed in early-stage GCs, indicating that these genes play a coordinating role in follicular recruitment and development. It is well known that *AMH* plays vital roles in follicle recruitment [27]. *HSD17B1* is a key enzyme for oestrogen synthesis, and *FST* promotes follicle development [28, 29]. Consistent with our results, Li et al. [15] recently found that these dynamic genes were also highly expressed in the early-stage by revealing the transcriptomic patterns of GCs at different follicle development stages in goats. However, the expression of these GC functional genes was significantly downregulated in ovarian aging. This study offers insights into the age-related molecular mechanisms underlying ovarian aging.

In addition, we described a set of transcription factors and their regulatory networks in GCs. Among these activated transcription factors, *FOXO1*, *SOX4*, and *HIF1A* are closely linked to ovarian aging. *FOXO1* changes in response to cellular stimulation, and is widely involved in cell proliferation, oxidative stress and apoptosis [30–32]. The current study showed that *FOXO1* showed specifically high expression in GCs. The function of *FOXO1 *depends on the modulation of downstream targets such as *ASB9*, *RUNX2*, *CEP55* and *STC1*, which are associated with proliferation in GCs. Moreover, *SOX4* is an important developmental transcription factor that plays important roles in stemness, differentiation, progenitor development, and multiple developmental pathways including PI3K, Wnt, and TGF-beta signalling [33]. Indeed, we observed the highest expression of *SOX4* in oocytes, and it was also abundant in GCs. Consistent with previous reports, we found that *SOX4* downstream target genes including *WNT5A*, *IGFBP2*, and *MARCKS*, are key regulatory elements of Wnt, TGF-beta and PI3K signalling in GCs. Notably, *SOX4* exhibited peak expression in aged ovaries, suggesting that *SOX4* may respond to ovarian aging. Moreover, *HIF1A* functions as a master transcriptional regulator of the adaptive response to hypoxia [34]. However, its role in the ovaries is less well understood. We found that *HIF1A* was specifically highly expressed in GCs. Furthermore, by comparing the expression levels between young and aging goats, *HIF1A* was downregulated in ovarian aging. Interestingly, *HIF1A* was not present in the atretic GCs of newborn goats. Network analysis revealed that *HIF1A* was strongly correlated with the downstream target gene *FST*. This is consistent with the result that the dynamics gene *FST* is involved in follicle development identified by pseudotime trajectory analysis. These genes we identified could be used as biomarkers and targets for the diagnosis and treatment of age-related ovarian disease.

## Conclusions

In summary, the present study provides the first comprehensive single-cell transcriptomic map of newborn, young, and aged goat ovaries and broadens our understanding of cell identities and age-related gene-expression alterations in GCs. For the first time, the study distinguished differences in the developmental trajectories and expression patterns of subcluster GCs in goats. Importantly, this study revealed the molecular mechanism of ovarian aging in goats and laid a foundation for evaluating the reproductive utilization years of goats. In addition, we identified a series of important genes or signalling pathways associated with ovarian aging at the single cell level, such as *FST*, *SOX4*, *HIF1A* and Wnt, Myc target signalling pathways, providing new targets for improving ovarian function in goats.

### Supplementary Information

**Additional file 1: Table S1.** List of marker genes used to score the cell cycle phases for each single cell.

**Additional file 2: Fig. S1.** Clustering analysis of various ovarian cells in goats. **A** Scatter plots showing the percentages of the nFeature, nCount, hemoglobin, mitochondrion, and ribosome genes expressed in goat ovaries. **B** The total UMIs and total genes were visualized by t-SNE. **C** The elbow plot shows standard deviations of the top 30 principal components in PCA. **D** A total of 23 clusters were visualized by t-SNE, and each point corresponding to a single cell is colour-coded according to its cluster membership. **E **The heatmap shows the different expression patterns of the top 10 characteristic genes in 23 clusters in the entire dataset. The expression level of each gene, from low to high, is indicated by a gradient from purple to yellow. **F** The dot plot shows the top 3 distinct expression patterns of the selected signature genes for each cluster. The specific gene expression levels and percentages of each cluster and sample are indicated by colour and dot size, respectively.

**Additional file 3: Tables S2–S4.** The barcode information of the newborn group, the young group and the aging group, respectively.

**Additional file 4: Table S5.** The recognition results of cellRanger cell.

**Additional file 5: Fig. S2.** Violin plots visualize the marker gene expression in cell Clusters 1–23. The specific gene expression levels and percentages of each cluster are indicated by colour and dot size, respectively.

**Additional file 6: Fig. S3.** Immunostaining of growing and atretic follicles for *AMH.* Scale bars = 100 μm.

**Additional file 7: Fig. S4.** Violin plots showing the expression levels of dynamic genes of GC subtypes in newborn, young and aging goats.

**Additional file 8: Table S6.** The significant regulons that regulated ovarian gene expression patterns.

**Additional file 9: Table S7.** The target genes of *HIF1A*, *SOX4*, and *FOXO1*.

## Data Availability

The ovarian scRNA-seq raw data of goats used in this study were publicly accessible at the National Center for Biotechnology Information (BioProject ID: PRJNA1010653).

## Abbreviations

ALPL: Alkaline phosphatase
AMH: Anti-Mullerian hormone
AUC: Area under the curve
BSA: Bovine serum albumin
C1QA: Complement C1q A chain
CD24: CD24 molecule
CD52: CD52 molecule
CD69: CD69 molecule
CDH5: Cadherin 5
COL1A1: Collagen type I alpha 1 chain
CRABP2: Cellular retinoic acid binding protein 2
CTSW: Cathepsin W
CYP11A1: Cytochrome P450 family 11 subfamily A member 1
CYP17A1A: Cytochrome P450 family 17 subfamily A member 1A
DAPL1: Death associated protein like 1
DCN: Decorin
DEGs: Differentially expressed genes
DRB3: HLA class II histocompatibility antigen, DRB1-4 beta chain
DSP: Desmoplakin
ECRG4: ECRG4 augurin precursor
EMX2: Empty spiracles homeobox 2
FBS: Fetal bovine serum
FIGLA: Folliculogenesis specific bHLH transcription factor
FOXO1: Forkhead box O1
FSHR: Follicle stimulating hormone receptor
FST: Follistatin
GCs: Granulosa cells
GEM: Gel Bead-in-emulsion
GNAS: Guanine nucleotide-binding protein G(s) subunit alpha
GO: Gene Ontology
GSEA: Gene set enrichment analysis
GSTM3: Glutathione S-transferase mu 3
GSVA: Gene set variation analysis
HIF1A: Hypoxia inducible factor 1 subunit alpha
HIGD1B: HIG1 hypoxia inducible domain family member 1B
HSD17B1: Hydroxysteroid 17-beta dehydrogenase 1
IGFBP5: Insulin like growth factor binding protein 5
INHA: Inhibin subunit alpha
KCNK12: Potassium two pore domain channel subfamily K member 12
KRT19: Keratin 19
KRT8: Keratin 8
LMO2: LIM domain only 2
MYBPC2: Myosin binding protein C2
MYH11: Myosin heavy chain 11
PBS: Phosphate buffered saline
PCA: Principle component analysis
PCs: Principal components
PDGFRA: Platelet derived growth factor receptor alpha
PECAM1: Platelet and endothelial cell adhesion molecule 1
PGRMC1: Progesterone receptor membrane component 1
PRDM1: PR/SET domain 1
PTPRC: Protein tyrosine phosphatase receptor type C
PTPRD: Protein tyrosine phosphatase receptor type D
RGS5: Regulator of G protein signaling 5
RT: Room temperature
SCENIC: Single-cell regulatory network inference and clustering
scRNA-seq: Single-cell RNA sequencing
SOX18: SRY-box transcription factor 18
SOX4: SRY-box transcription factor 4
SPON2: Spondin 2
STC1: Stanniocalcin 1
TAC1: Tachykinin precursor 1
TAGLN: Transgelin
TFs: Transcription factors
THBS1: Thrombospondin 1
TIMP1: TIMP metallopeptidase inhibitor 1
t-SNE: t-Distributed Stochastic Neighbor Embedding
UMAP: Uniform Manifold Approximation and Projection
UMI: Unique molecular identifier
