# Supplementary figures and images for: Single-cell transcriptomic atlas of goat ovarian aging
Source: J Anim Sci Biotechnol. 2023 Dec 6;14:151. doi: 10.1186/s40104-023-00948-8 (PMC10699009; doi:10.1186/s40104-023-00948-8)

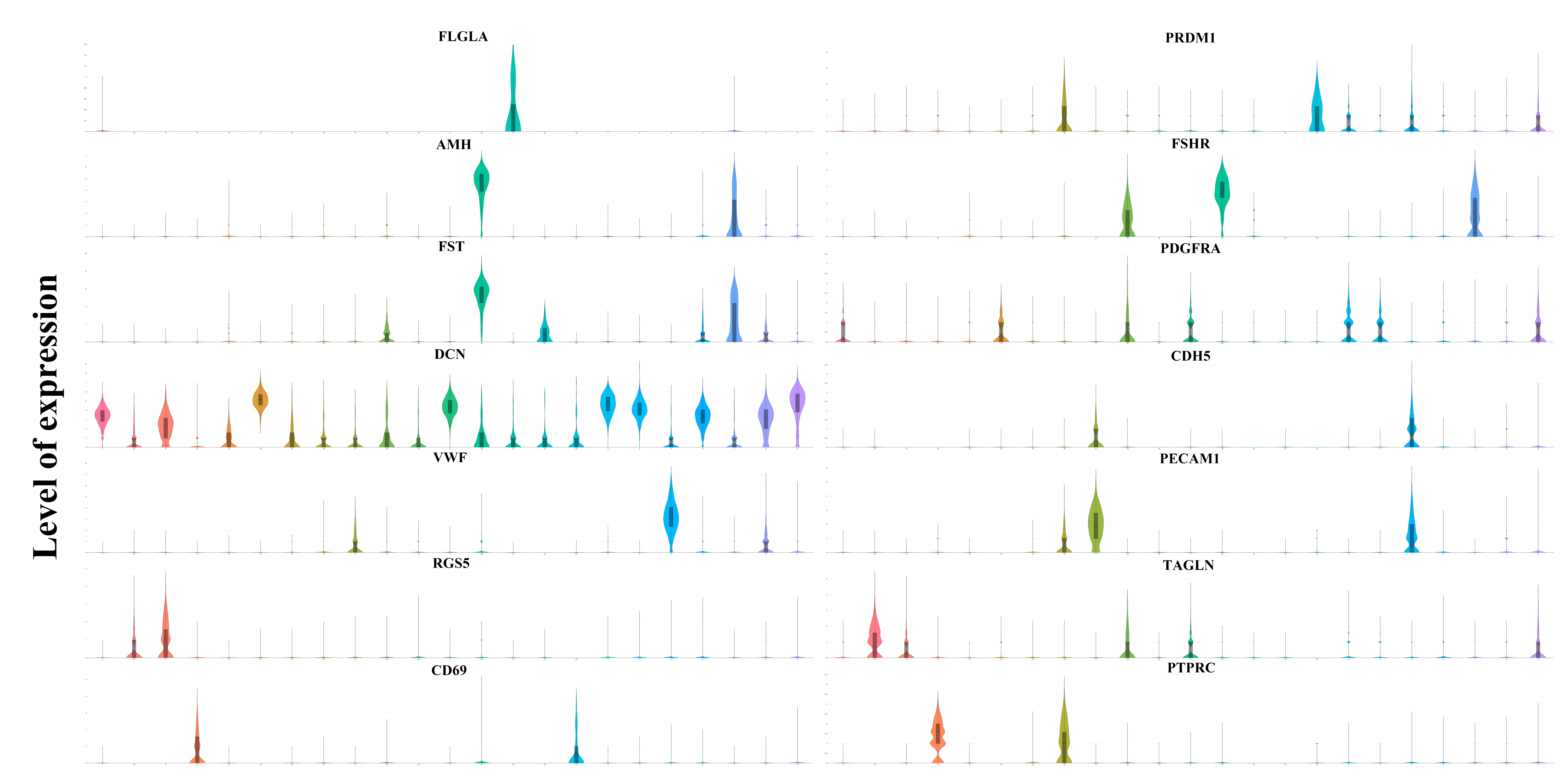

Supplement: Supplementary file 5 — Additional file 5: Fig. S2. Violin plots visualize the marker gene expression in cell Clusters 1–23. The specific gene expression levels and percentages of each cluster are indicated by colour and dot size, respectively. [file 40104_2023_948_MOESM5_ESM.png]

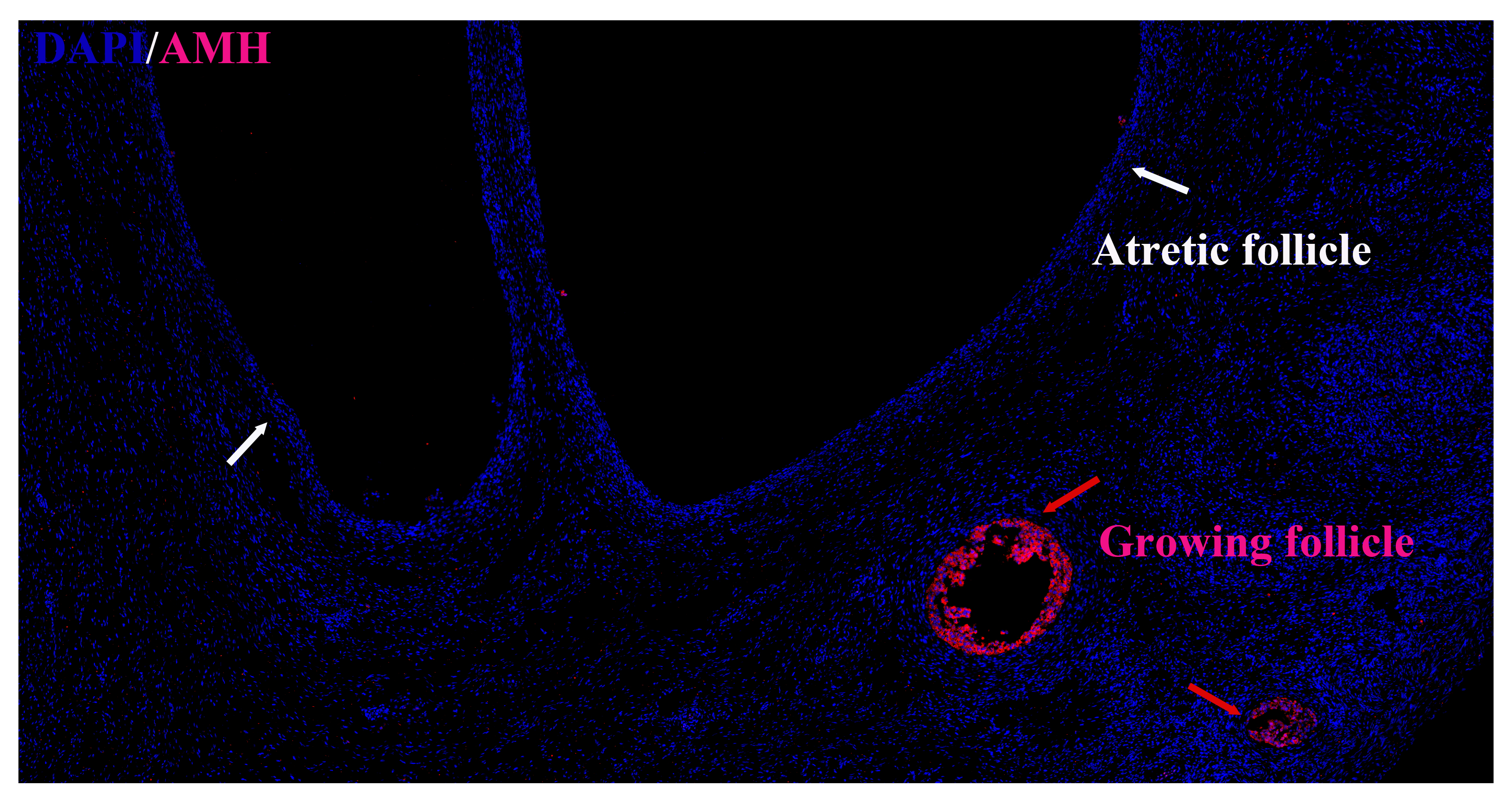

Supplement: Supplementary file 6 — Additional file 6: Fig. S3. Immunostaining of growing and atretic follicles for AMH. Scale bars = 100 μm. [file 40104_2023_948_MOESM6_ESM.tif]

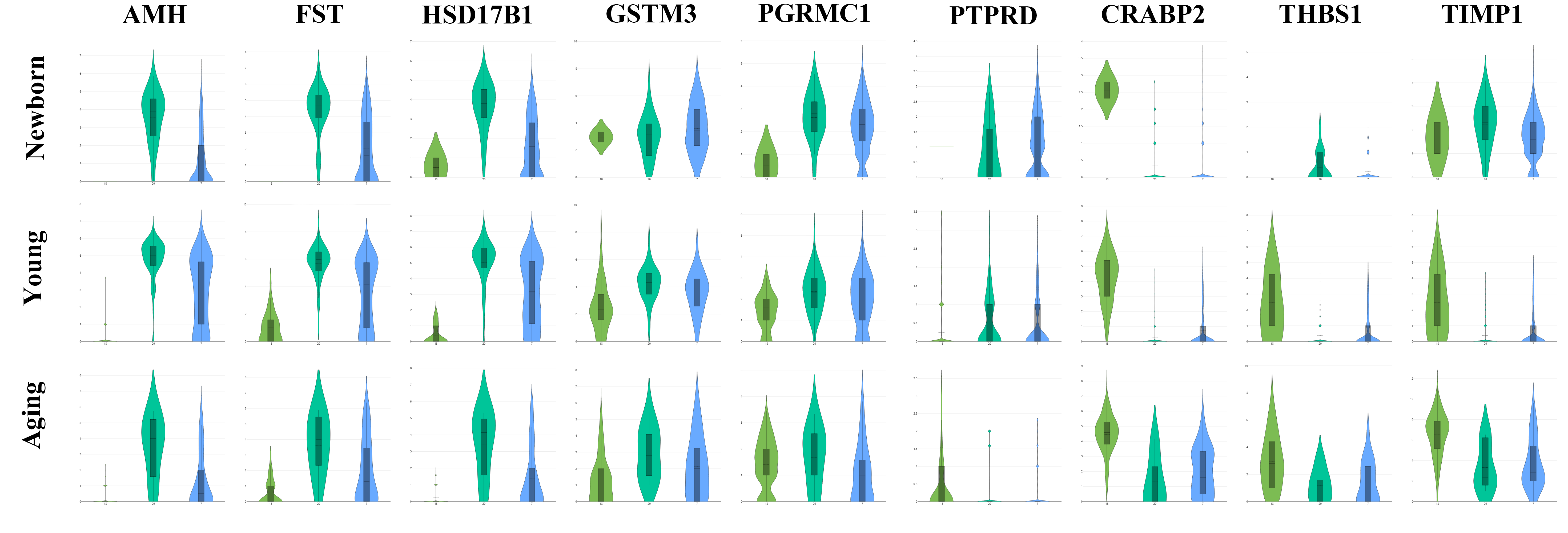

Supplement: Supplementary file 7 — Additional file 7: Fig. S4. Violin plots showing the expression levels of dynamic genes of GC subtypes in newborn, young and aging goats. [file 40104_2023_948_MOESM7_ESM.png]
